# A degradation-based sorting method for lithium-ion battery reuse

**DOI:** 10.1371/journal.pone.0185922

**Published:** 2017-10-12

**Authors:** Hao Chen, Julia Shen

**Affiliations:** 1 School of Automotive Engineering, Shanghai University of Engineering Science, Shanghai, P.R. China; 2 Department of Computer and Information Science, University of Michigan-Dearborn, Dearborn, MI, United States of America; 3 Detroit Country Day, Beverly Hills, United States of America; Beihang University, CHINA

## Abstract

In a world where millions of people are dependent on batteries to provide them with convenient and portable energy, battery recycling is of the utmost importance. In this paper, we developed a new method to sort 18650 Lithium-ion batteries in large quantities and in real time for harvesting used cells with enough capacity for battery reuse. Internal resistance and capacity tests were conducted as a basis for comparison with a novel degradation-based method based on X-ray radiographic scanning and digital image contrast computation. The test results indicate that the sorting accuracy of the test cells is about 79% and the execution time of our algorithm is at a level of 200 milliseconds, making our method a potential real-time solution for reusing the remaining capacity in good used cells.

## Introduction

The battery is an electrochemical cell that can be charged electrically to provide electric power as needed, and the rechargeable battery is a secondary cell that can store excess energy from renewable energy sources. Owing to circumstances in recent years ranging from the increasing demand for electric vehicles to air pollution by burning gasoline, use of the lithium-ion battery (a common type of rechargeable batteries) is on the rise. Based on a study performed by Navigant Research, the worldwide revenue of Lithium-ion (Li-ion) cells [1] is expected to reach $26 billion US dollars in 2023 due to its wide applications in hybrid/electric automotive, commercial vehicles, aerospace, military operations, and home electronics.

There have, however, been numerous concerns with respect to the availability of Lithium—an element crucial to the production of Li-on batteries. Because Lithium recycling is relatively new, it is underdeveloped and has not yet been economically feasible. The current processing activities at recycling facilities [2] and in academic studies [3–6] are limited to the cell destruction [7] for recovering chemical elements in batteries. The three shortcomings of the cell destruction are

1. *restricted accessibility*: only few facilities are available worldwide for the element recovery;
2. *economic non-viability*: the cost of the element recovery is higher than that of mining the elements; and
3. *waste of resources*: the undifferentiated destruction destroys both good and bad cells.

Since the commercial values of a repurposed rechargeable battery and the element recovery of the cell are about $2.5 and $0.5 per cylindrical cell (Model 18650) respectively, each repurposed cell creates a $2 extra value, compared to the element recovery.

We completed a comprehensive search on existing methods via different library and online tools, including Google Scholar, Engineering Village, and over 30 engineering, scientific & technical databases (such as ACM digital library, IEEE Xplore and INSPEC) at the University of Michigan library. Our search results indicate that there is no similar research paper or patent on the real-time, degradation-based sorting methodology. Few studies [8–10] have been carried out on mathematical models to predict the degradation of Li-ion batteries rather than a sorting method based on measuring the degradation of batteries. Some other activities were related to the mechanical behaviors of batteries [11, 12].

In the field of material damage, various sensing technologies have been used in the past, including ultrasonic wave propagation [13–15], vibration [16], acoustic emission [17], and X-ray computed tomography [18–21]. But, X-ray measurement has not been applied in quantifying the degradation of Lithium-ion batteries.

In addition, the existing methods are far too slow in separating good cells from bad cells. The measurement of battery capacity is involved with a charging and discharging process, which takes several hours to complete; the measurement of internal resistance in battery cells requires contact measurement, which is not ideal for a fast-moving conveyor belt at battery recycling facilities. It is also nearly impossible to measure the internal resistance of each cell inside a laptop battery pack without breaking the case. The combination of all the shortcomings of the entire procedure makes its automation in real time both difficult and entirely unsuitable. In a recycling facility where tens of thousands of Li-ion cells need to be processed every day, sorting all the battery cells in an efficient way is one of the primary obstacles to be overcome in the repurposed battery market.

In 2015 alone, the annual worldwide production capacity of 18650 batteries was about 3 billion cells (Avicenne Energy [1]), indicating a potential market for the battery reuse. The objective of this study is to develop a new method for sorting 18650 Lithium-ion (Li-ion) batteries in large quantities and in real time at battery recycling facilities to harvest used cells with enough remaining capacity.

The significant benefits of our new method include (a) the first nondestructive real-time method for diagnosing battery degradation via X-ray imaging, (b) a new way for the lithium battery recycling process with an economic benefit from the repurposed batteries, and (c) energy conservation through our sorting process that reduces the need to produce new Li-on batteries.

The rest of this paper is organized as follows. In Section 2, test materials and instruments are introduced and in Section 3 computational schemes are presented for estimating the battery degradation based on digital radiographic images. Experimental and computing results are compared in Section 4, followed by some concluding remarks in Section 5.

## Materials & instruments

**Fig 1:**
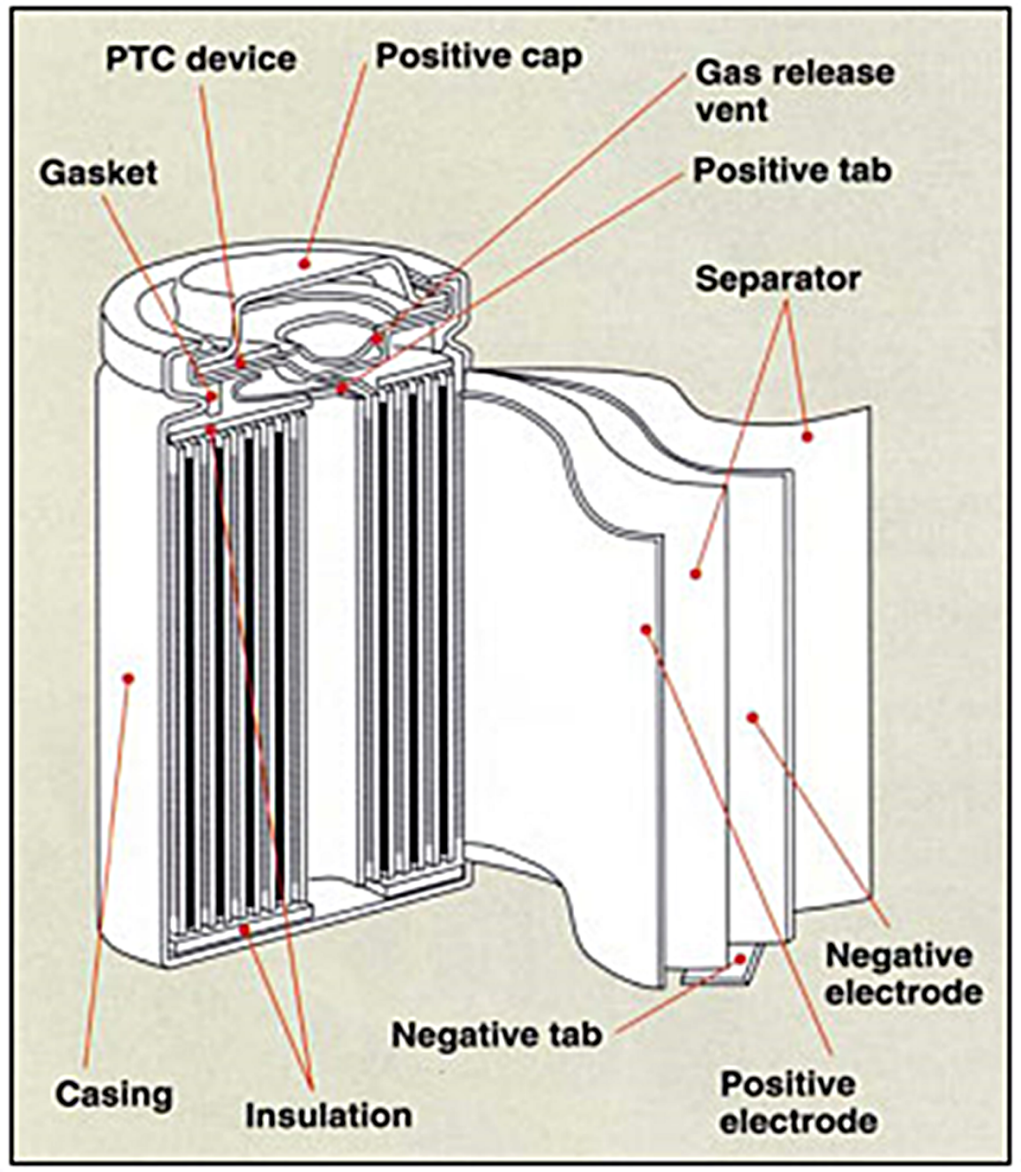
Internal structures of Li-ion battery. Source: Sanyo.

**Fig 2:**
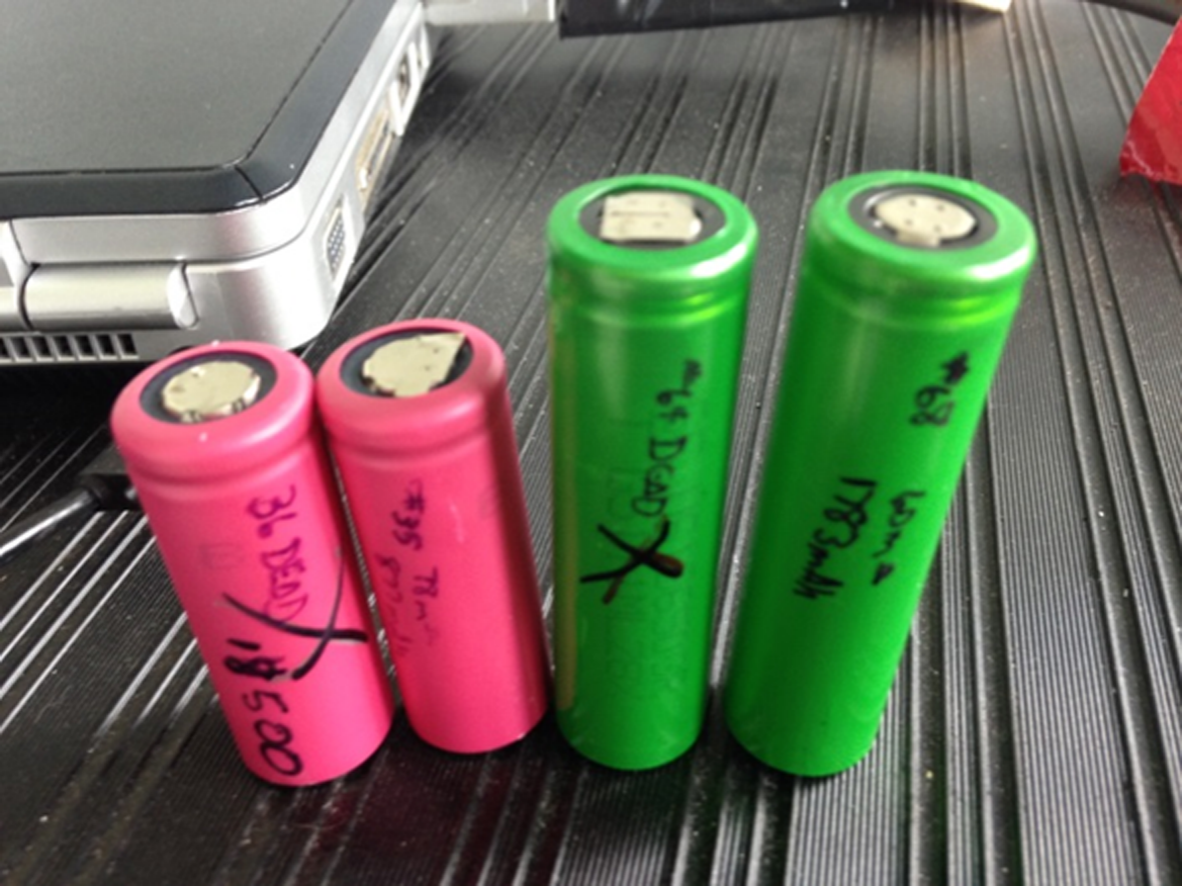
18650 Li-ion batteries.

The Lithium-ion (Li-ion) battery is a type of rechargeable batteries in which lithium ions move from a negative electrode to a positive electrode during a discharging process and in the reverse direction during a charging process, as shown in Fig 1. Owing to its high energy density and small memory effect, the Li-ion battery has been extensively used in a range of home electronics. Here, the high energy density indicates the high energy capacity that this battery can store with respect to the same cell volume, and the small memory effect means that this battery can be recharged at any current charging degree without losing the maximum energy capacity. 18650 Li-ion battery (18mm in diameter and 65mm in length) is one of the most common cylindrical types of rechargeable batteries used in laptops (6 to 12 cells per pack) and Tesla electric cars (7,104 cells in one model S), as shown in Fig 2.

**Fig 3:**
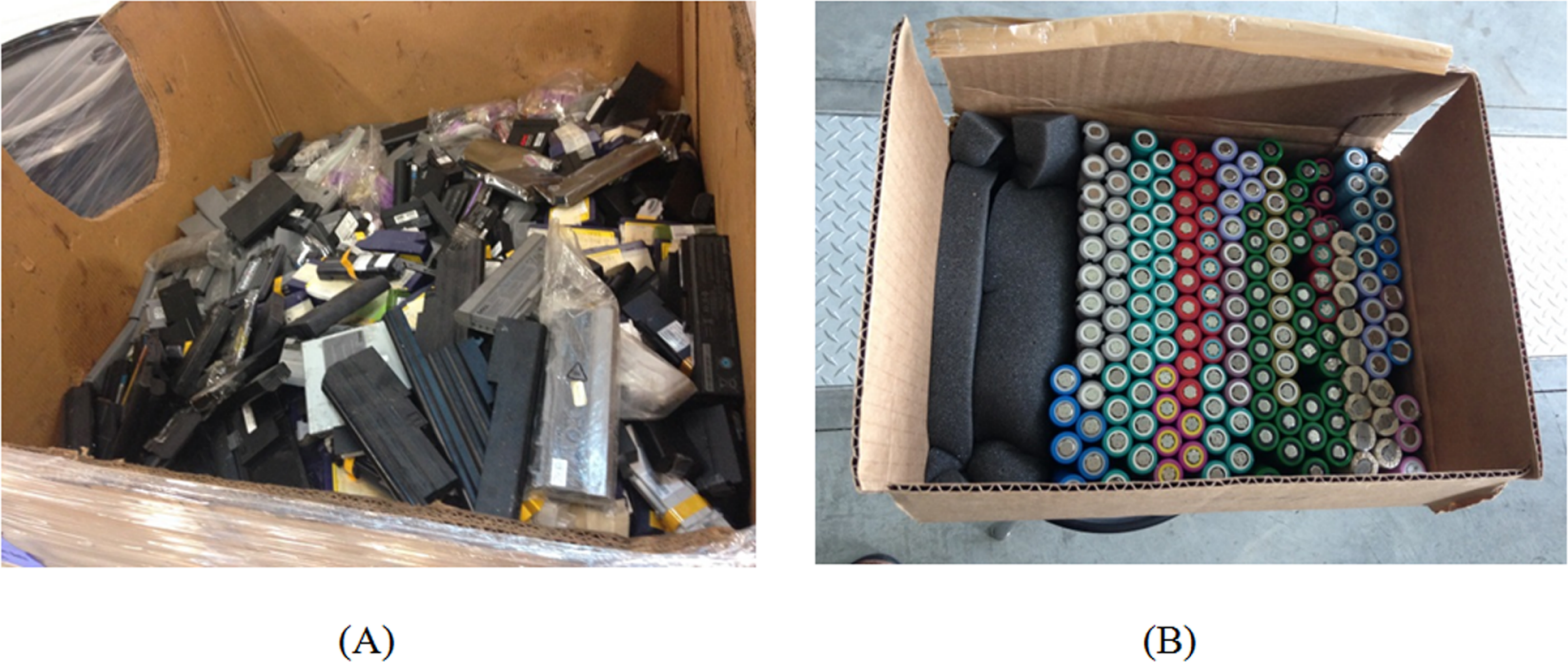
Batteries to be recycled. (A) Cell inside packs. (B) Test cells in this study.

Although the Li-ion battery does not contain lead or cadmium, most states in U.S.A. generally advise against their disposal in landfills. The elements (iron, copper, nickel and cobalt) of Li-ion batteries can, however, be recycled [22], as shown in Fig 3. Even so, the process in not viable economically, as the recycling cost is more expensive than the actual cost of mining the elements. There are also only few recycling facilities available worldwide for the element recovery. Consequently, in many countries the element recovery can’t be accomplished unless the batteries are shipped overseas.

**Fig 4:**
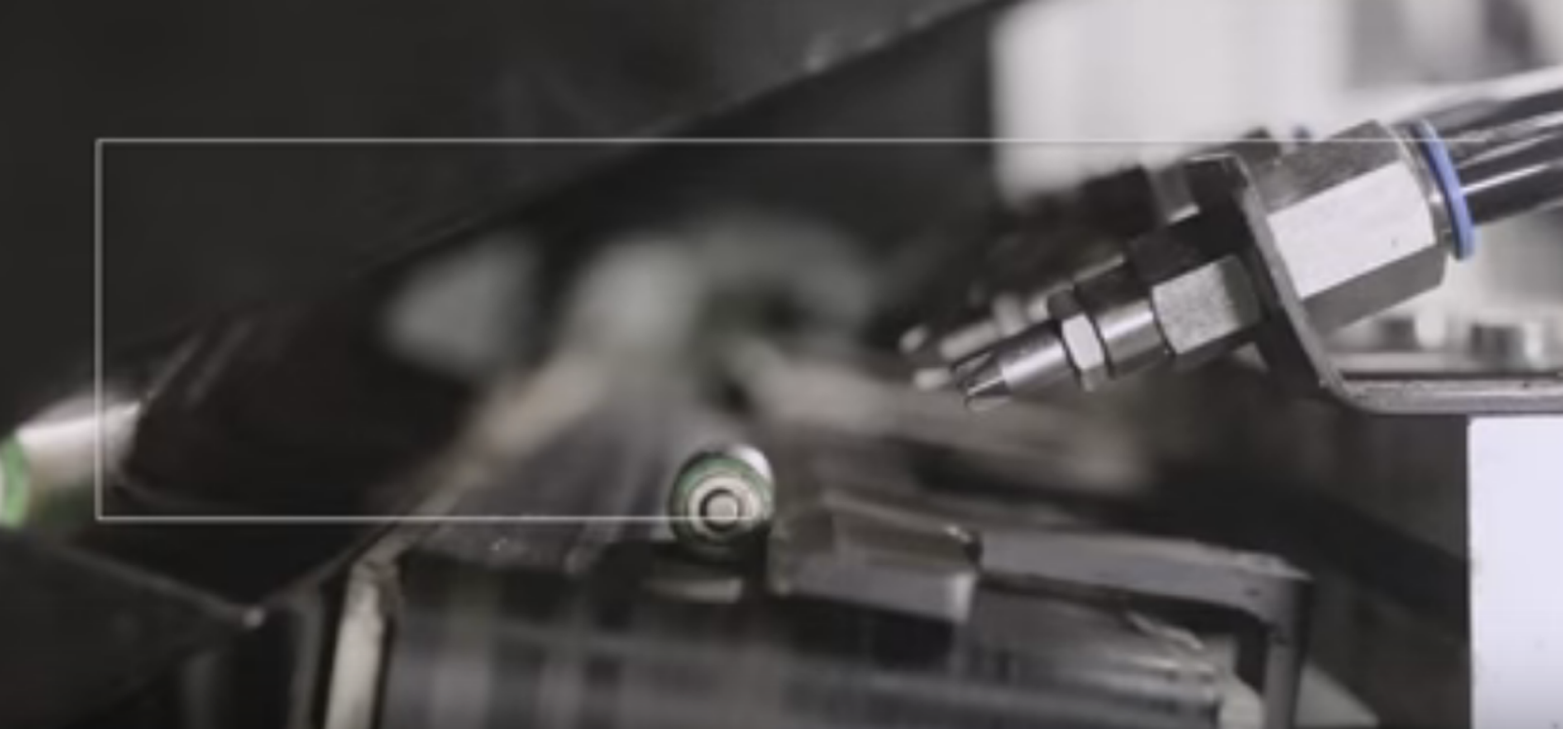
OBS 600 –Automatic sorting machine for waste portable batteries. Source: Optisort.

One group of existing studies were focused primarily on the optical sorting [23] of batteries, in which the batteries were sorted into different types based on their external shapes and appearances, as illustrated in Fig 4. Another group of methods were based on the measurement of electric conditions [24–26] of the batteries via contact sensing, which is difficult to be executed in a real-time automatic process. Essentially, no previous research was found regarding the swift sorting of batteries into good and bad cells based on the remaining useful life caused by battery degradation.

The materials used in this study include

**Fig 5:**
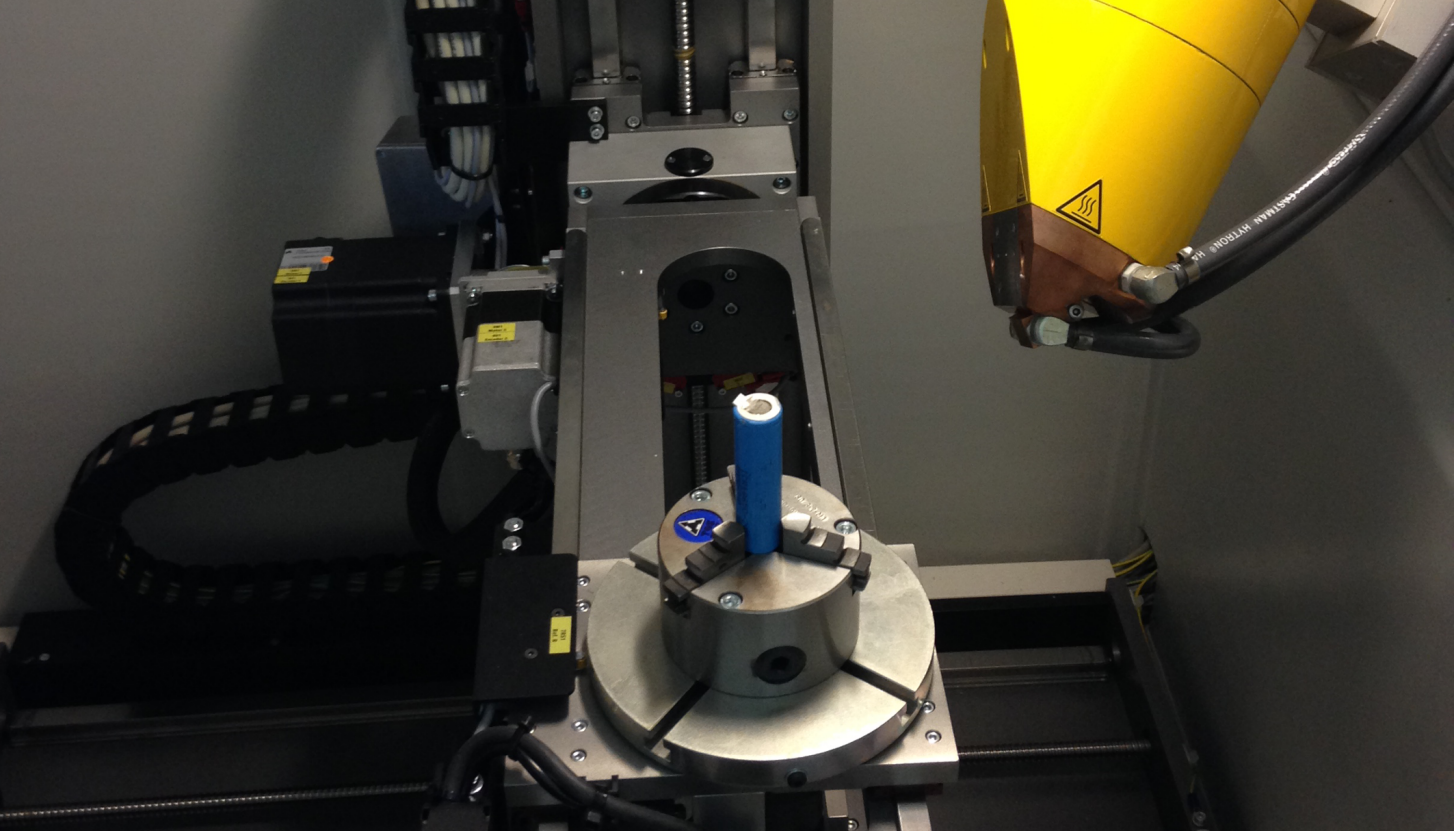
A micro-focused X-ray radiographic system at the University of Michigan.

1. 58 used 18650 Li-ion batteries that were obtained from a local battery recycling company, as shown in Fig 3(B); and
2. 174 X-ray radiographic images. Each cell was scanned three times in a micro-focus X-ray CT system (Fig 5) at three different angles with an interval of 120°. Scanning resolution is between 10 to 20 micrometers.

**Fig 6:**
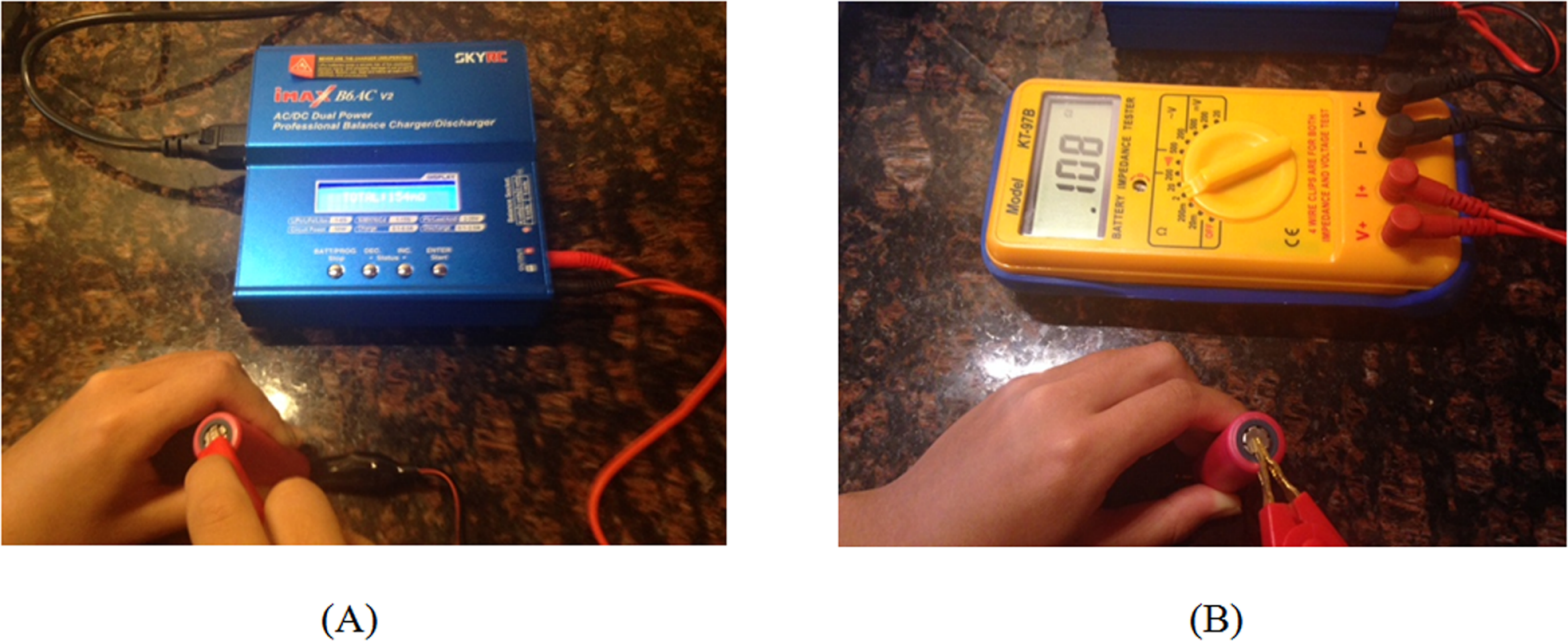
Test instruments in this study. IMAX B6AC Charger/Discharger. (B) KT-97B battery impedance tester.

The following instruments and software tools were used: (a) MATLAB software (especially the image processing toolbox), (b) A SkyRC B6AC charger/discharger (Fig 6(A)), (c) A KT-97B battery impedance tester (Fig 6(B)), and (d) An ASUS laptop (Model: N56V; Memory 6G; CPU 2.3GHz).

In order to validate the sorting accuracy of our method, the following two properties of batteries are considered in this paper.

### Battery capacity

Capacity is an index for the health of 18650 Li-ion cells and it is quite difficult to measure in real time. In this study, the traditional charge/discharge/charge cycle was used to measure the current capacity of the test cells via an IMAX B6AC Charger/Discharger and a custom-built instrument. The original capacity of the test cells was obtained from the product information.

### Internal resistance of battery

Internal resistance provides information on the remaining useful life of battery cells. The higher the internal resistance is, the closer the cell approaches to the end of life. In this study, a KT-97B battery impedance tester and/or a custom-built instrument were used for measuring the internal resistance of the test cells.

## Computational methods

**Fig 7:**
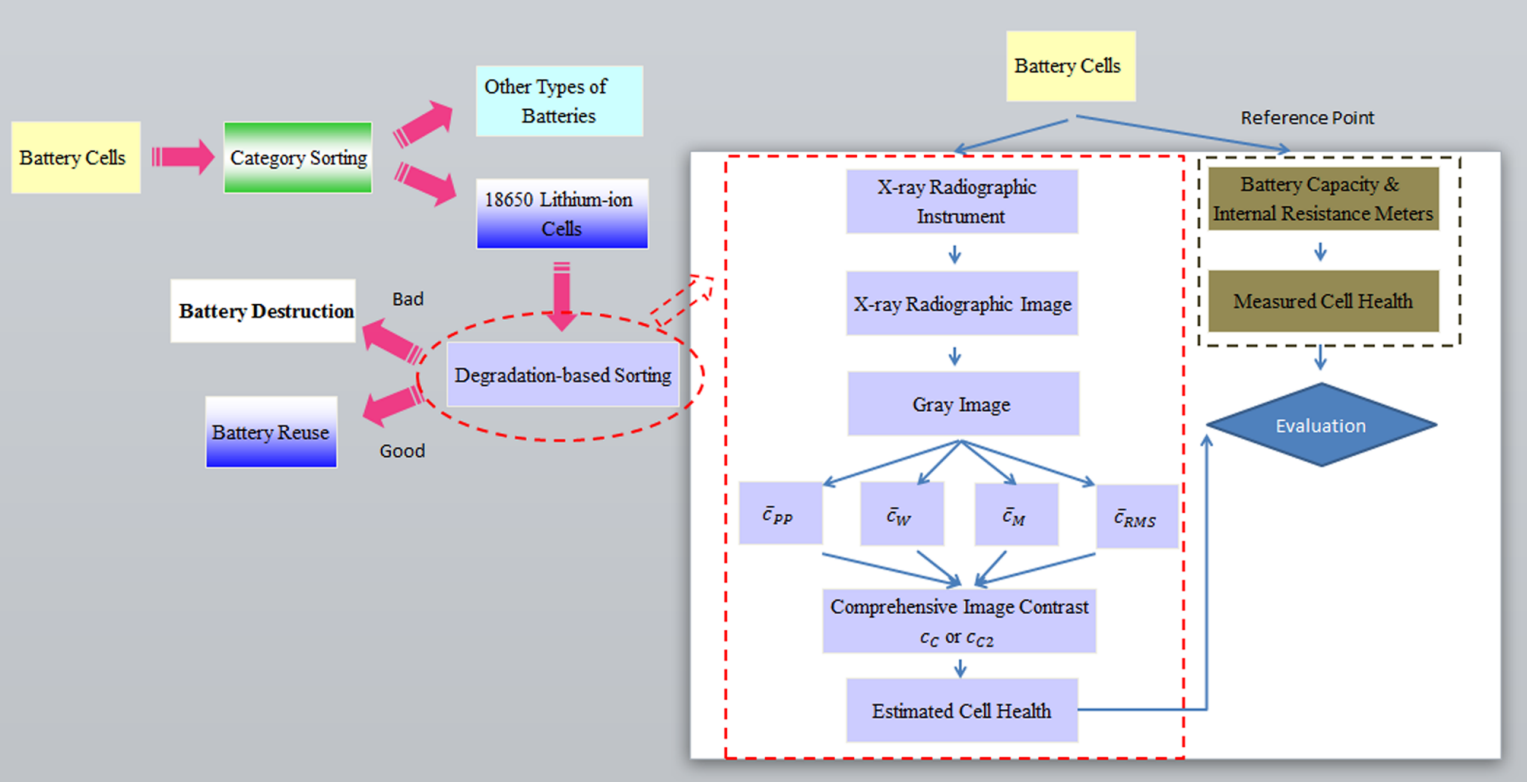
Application and design of degradation-based sorting in battery recycling.

The left part of Fig 7 provides a diagram about how our degradation-based sorting will be used in a complete battery recycling process. Note that the category sorting is executed by the traditional optical scanning. The design on the degradation-based method is carried out in an approach described in the right part of Fig 7, in which the cell health means the health status (i.e., the opposite of degradation) of battery cells. The measured health of the cells is observed by using the battery capacity and internal resistance meters, while the X-ray images of cells are processed to determine the computed health of cells. The closeness between the measured and the computed health values validates the feasibility of developing our sorting method.

The flowchart of our degradation-based method is provided in the right portion of Fig 7, and the details of each component are described below.

**Fig 8:**
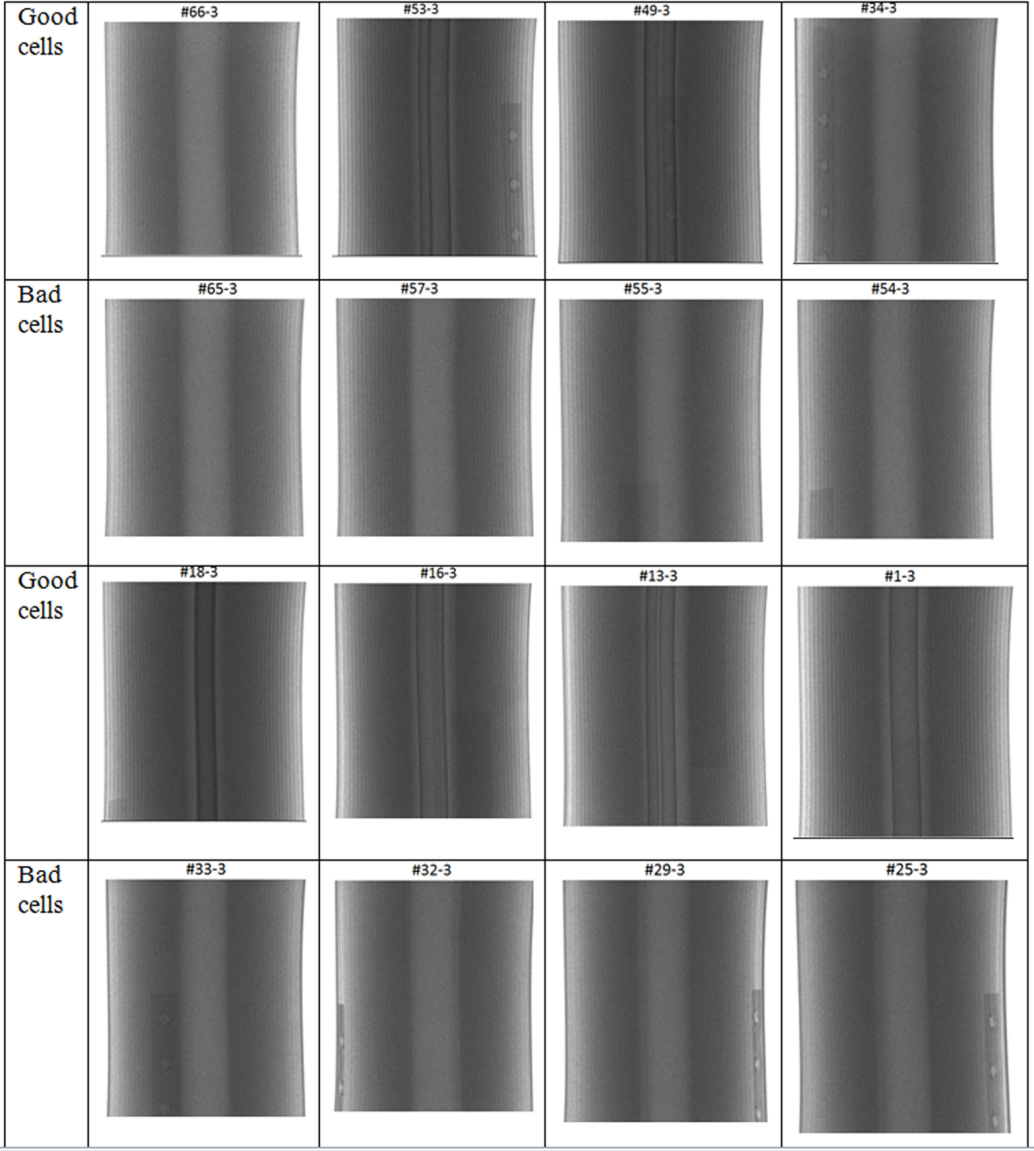
Comparison between X-ray radiographic images of the good and bad battery cells.

After our careful examination of all 174 X-ray images, we found that the image contrast of the internal structures of the battery cells was a good indicator to represent the health or degradation of the battery cells. The good cells generally have a clearer internal structure than the bad cells, as illustrated in Fig 8.

**Fig 9:**
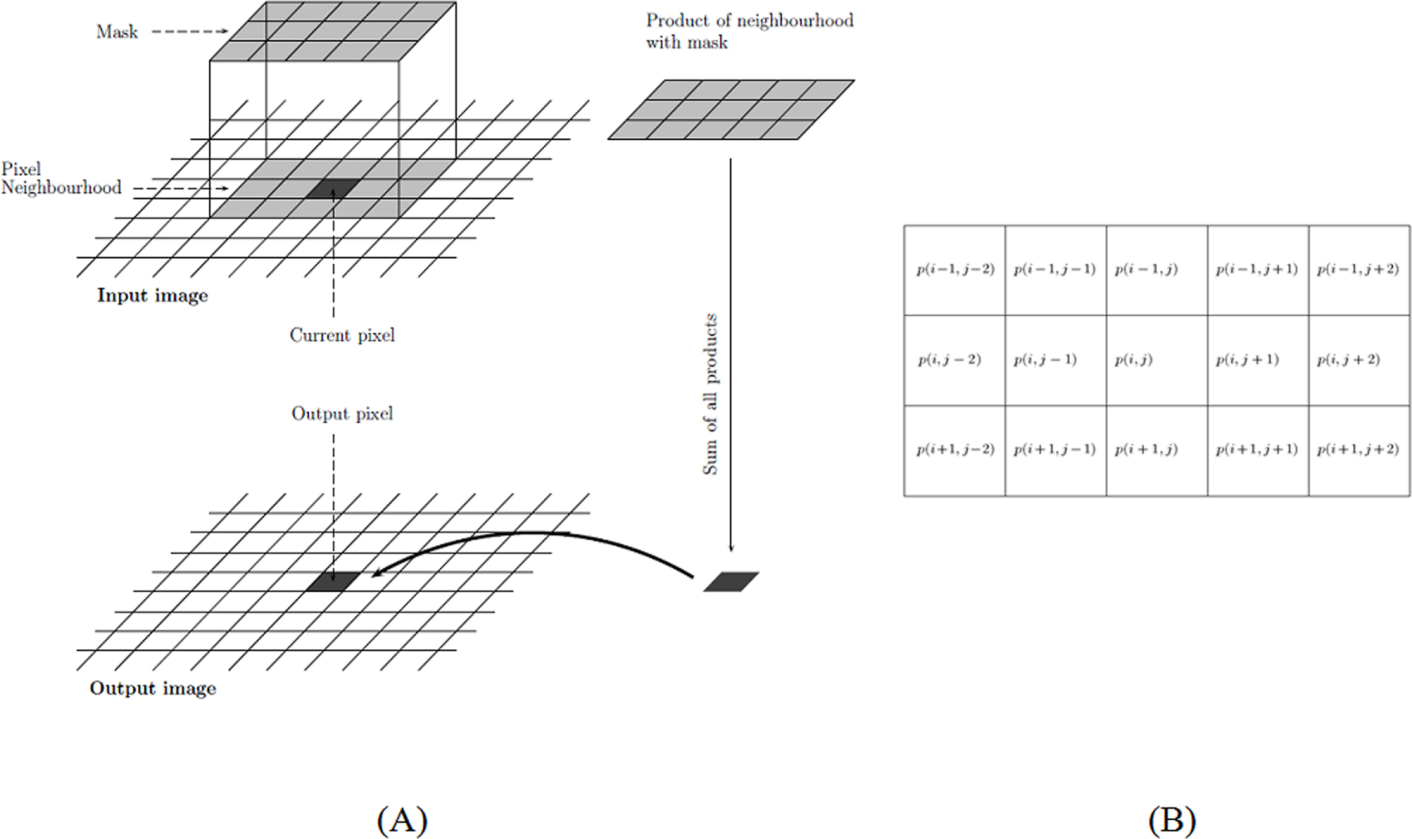
Notation of a digital image (Source: Alasdair McAndrew). (A) A mask as a neighborhood of current pixel with respect to input and output images. (B) Current pixel (i, j) and its neighboring pixels.

Both local and global analyses are considered to quantify the image contrast of the internal structures of the battery cells. A local analysis utilizes only image information in a local neighborhood, while in a global analysis the information over an entire image is used to compute a local feature. Let I(*i*, *j*) and c(*i*, *j*) be the intensity and contrast values at pixel (*i*, *j*), as illustrated in Fig 9, where *i* ∈ [1,*m*] and *j* ∈ [1,*n*] represent rows and columns of the image, respectively. The intensity values are represented in a range [0.0, 1.0], where 0.0 and 1.0 refer to black and white colors, respectively.

### Local analysis

Based on image processing [14], the first index, *c*_1_(*i*,*j*), is defined as an accumulative difference in the intensities between the current pixel and one ring of neighboring pixels (Fig 9):

$$c_{1}(i,j)=9I(i,j)-\sum_{x=i-1}^{i+1}\sum_{y=j-1}^{j+1}I(x,y)\;i=1,m,\;j=1,n.$$ (1)

In the boundary cases in which *x* ∉ [1,*m*] or *y* ∉ [1,*n*], the calculation of *I*(*x*,*y*) is skipped.

The overall *c*_1_ for the entire image is

$${\bar{c}}_{1}=\frac{1}{nm}\sum_{i=1}^{m}\sum_{j=1}^{n}|c_{1}(i,j)|.$$ (2)

The second index, *c*_2_(*i*,*j*), is defined as a ratio of the difference between the local maximum and minimum to the sum of local maximum and local minimum [27]

$$c_{2}(i,j)=\frac{I_{max}(i,j)-I_{min}(i,j)}{I_{max}(i,j)+I_{min}(i,j)},$$ (3)

where,

$$I_{max}(i,j)=\arg{max}_{x\in [i-k,i+k],y\in [j-k,j+k]}\;I(x,y),$$ (4)

$$I_{min}(i,j)=\arg{min}_{x\in [i-k,i+k],y\in [j-k,j+k]}\;I(x,y),$$ (5)

*k* is a positive integer that represents the half size of a pixel neighborhood.

The overall *c*_2_ contrast for the entire image is

$${\bar{c}}_{2}=\frac{1}{nm}\sum_{i=1}^{m}\sum_{j=1}^{n}c_{2}(i,j).$$ (6)

### Global analysis

The first global index in this paper is defined as

$$c_{3}(i,j)=\frac{\left|I(i,j)-I_{b}\right|}{I_{b}},$$ (7)

where *I*_b_ *I*_*b*_ is the intensity value of background and equals 1.0 in this study. Since *I*_b_ is always greater than or equal to *I*(*i*, *j*) here, |*I*(*i*,*j*)-*I*_b_| can be replaced by *I*_b_-*I*(*i*,*j*).

The overall *c*_3_ contrast for the entire image is

$${\bar{c}}_{3}=\frac{1}{nm}\sum_{i=1}^{m}\sum_{j=1}^{n}c_{3}(i,j).$$ (8)

Inspired by the root mean square contrast (RMS) [28], the fourth index is defined as

$${\bar{c}}_{4}=\sqrt{\frac{1}{nm}\sum_{i=1}^{m}\sum_{j=1}^{n}{\left[I(i,j)-\bar{I}\right]}^{2}},$$ (9)

where,

$$\bar{I}=\frac{1}{nm}\sum_{i=1}^{m}\sum_{j=1}^{n}I(i,j).$$ (10)

### Hybrid analysis

We define a composite index for image contrast, *c*_1234_, as a linear combination of the above four different indices:

$$c_{1234}=k_{1}{\bar{c}}_{1}+k_{2}{\bar{c}}_{2}+k_{3}{\bar{c}}_{3}+k_{4}{\bar{c}}_{4},$$ (11)

$$k_{1}+k_{2}+k_{3}+k_{4}=1.0,$$ (12)

where *k*_1_, *k*_2_, *k*_3_, *k*_4_ are weights subject to the constraint in Eq (12). In this paper, 0.25 is assigned to each of these weights. We also define another composite index:

$$c_{34}=q_{3}{\bar{c}}_{3}+q_{4}{\bar{c}}_{4},$$ (13)

$$q_{3}+q_{4}=1.0,$$ (14)

in which both *q*_3_ and *q*_4_ are assigned to be 0.5.

## Results of experiment and computation

We implemented the above formulas by means of the image processing toolbox in MATLAB, which is a software tool developed by MathWorks. The computer used in this study is an ASUS laptop computer with Intel i7 CPU@2.30GHz and 16 GB RAM.

### Test and computation results

In this study, we measured the internal resistance and current capacity of the 58 test batteries, as illustrated in Table 1. In this table, the battery capacity reduction, *bc*_r_, is defined as

$${bc}_{r}=\frac{{bc}_{o}-{bc}_{c}}{{bc}_{o}},$$ (15)

Where *bc*_o_ and *bc*_c_ are the original and current capacities of a battery cell, respectively.

**Table 1:** Capacity reduction ratio and internal resistance of test batteries (∞ refers to a resistance greater than 1 ohm.).

ID	Model	*bc*_*r*_	Internal Resistance (milliohm)	*Mhc*	*c*_*C*2_	*Chc*
1	Samsung ICR18650-26F	0.34	59.1	Good	0.3731	Good
2	Samsung ICR18650-26F	0.31	58.5	Good	0.3818	Good
3	Samsung ICR18650-26F	0.30	58.4	Good	0.379555	Good
4	Samsung ICR18650-24E	0.25	56.9	Good	0.34945	Bad
5	Samsung ICR18650-24E	0.28	56.9	Good	0.3618	Good
6	LG Chem LGDBB31865	0.12	44.2	Good	0.37195	Good
7	LG Chem LGDBB31865	0.12	43.1	Good	0.3742	Good
8	ATL IMR18650	0.4	64.7	Good	0.36315	Good
9	ATL IMR18650	0.09	60.2	Good	0.3645	Good
10	LG Chem LGDAS31865	0.11	63.2	Good	0.351565	Bad
11	LG Chem LGDAS31865	0.17	72.3	Good	0.3617	Good
12	LG Chem LGDAS31865	0.11	80.4	Good	0.35814	Good
13	LG Chem LGDAS31865	0.14	69.6	Good	0.36942	Good
14	LG Chem LGDAS31865	0.09	65.8	Good	0.3818	Good
15	LG Chem LGDAS31865	0.12	66.0	Good	0.3597	Good
16	LG Chem LGDAS31865	0.10	65.4	Good	0.3808	Good
17	LG Chem LGDAS31865	0.1	62.8	Good	0.37402	Good
18	Samsung ICR18650-28A	0.2	48.1	Good	0.39322	Good
19	Samsung ICR18650-28A	0.21	48.5	Good	0.39325	Good
20	Samsung ICR18650-28A	0.18	49.9	Good	0.4145	Good
21	Samsung ICR18650-28A	0.18	49.5	Good	0.3919	Good
22	Sanyo 4UR18650-3-3	1.0	∞	Bad	0.35705	Good
23	Sanyo 4UR18650-3-3	1.0	∞	Bad	0.35085	Bad
24	Sanyo 4UR18650-3-3	1.0	∞	Bad	0.34745	Bad
25	Sanyo 4UR18650-3-3	1.0	∞	Bad	0.36434	Good
26	Sanyo 4UR18650-3-3	1.0	∞	Bad	0.354	Bad
27	Sanyo 4UR18650-3-3	1.0	∞	Bad	0.35675	Bad
28	Sanyo 4UR18650-3-3	1.0	∞	Bad	0.347285	Bad
29	Sanyo 4UR18650-3-3	1.0	∞	Bad	0.355445	Bad
30	Sanyo 4UR18650-3-3	1.0	∞	Bad	0.359975	Good
31	Sanyo 4UR18650-3-3	1.0	∞	Bad	0.35146	Bad
32	Sanyo 4UR18650-3-3	1.0	∞	Bad	0.35182	Bad
33	Sanyo 4UR18650-3-3	1.0	∞	Bad	0.354945	Bad
34	Sanyo UR18500F	0.46	76.2	Good	0.3644	Good
35	Sanyo UR18500F	0.46	78.8	Good	0.38185	Good
36	Sanyo UR18500F	1.0	∞	Bad	0.361965	Good
37	Sanyo UR18500F	1.0	∞	Bad	0.360705	Good
38	Sanyo UR18500F	1.0	∞	Bad	0.34578	Bad
41	Sanyo UR18500F	1.0	∞	Bad	0.35161	Bad
42	Sanyo UR18650ZT	0.31	55.2	Good	0.387	Good
49	Sanyo UR18500F	0.33	59.7	Good	0.39045	Good
52	Sanyo UR18500F	0.36	58.1	Good	0.38505	Good
53	Sanyo UR18500F	0.32	59.8	Good	0.38735	Good
54	Sony US1860GR	1.0	∞	Bad	0.34765	Bad
55	Sony US1860GR	1.0	∞	Bad	0.3568	Bad
56	Sony US1860GR	1.0	∞	Bad	0.3405	Bad
57	Sony US1860GR	1.0	∞	Bad	0.3465	Bad
58	Sony US1860GR	0.75	161.3	Bad	0.34445	Bad
59	Sony US1860GR	0.79	164.7	Bad	0.35219	Bad
60	Sony US1860GR	0.26	157.2	Bad	0.3483	Bad
61	Sony US1860GR	0.29	156.1	Bad	0.3419	Bad
64	Sony US1860GR	1.0	∞	Bad	0.33495	Bad
65	Sony US1860GR	1.0	∞	Bad	0.33685	Bad
66	Sony US1860GR	0.18	63.9	Good	0.331	Bad
67	Sony US1860GR	0.19	57.8	Good	0.34525	Bad
68	Sony US1860GR	0.21	60.5	Good	0.3525	Bad
69	Sony US1860GR	0.19	58.2	Good	0.34225	Bad
70	Sony US1860GR	0.14	61.8	Good	0.34175	Bad

The capacity reduction ratio and internal resistance are used to infer the measured health of each cell, *mhc*: a good cell or a bad cell. A good 18650 cell is defined as a cell with an internal resistance smaller than 150 milliohms, while a bad 18650 cell is considered as a cell with an internal resistance of over 150 milliohms. Table 1 also includes the composite contrast index, *c*_34_, of Eq (13). Based on *c*_34_ and a chosen threshold *t* (0.357), the computed health of each cell, *chc*, can be provided in the following binary way:

$$Computed\;cell\;health=\left\{ \begin{array}{c} good,\;if\;c_{34}>t\\ bad,\;if\;c_{34}\leq t \end{array}\right..$$ (16)

In comparison between *mhc* and *chc*, the overall sorting accuracy of the test cells was about 79%. This means that 79% of the test cells were correctly sorted on the basis of their degradation status. An accuracy formula is defined as

$$accuracy=\frac{true\;positives+true\;negatives}{overall\;population}\;(\%),$$ (17)

where positives refer to the good cells and negatives mean the bad cells.

A more detailed distribution of false negative and false positive cases is given in Table 2. The execution time of the first 5 cells is given in Table 3. The time cost of the other cells is in a similar range, making our method fast enough for real-time applications.

**Table 2:** The distribution of true and false predictions of our model (positive: good cells; negative: bad cells).

	Positive cell	Negative Cell
True prediction	25	21
False prediction	7	5

**Table 3:** Execution time for computing *c*_c2_ of the first 5 cells (image size: 1128x1022 pixels).

Cell ID	1	2	3	4	5
Time (second)	0.23	0.23	0.23	0.22	0.22

Since both *c*_3_ and *c*_4_ are independent upon the size of mask, there is only one unique *c*_34_ value for each X-ray radiographic image, compared to *c*_1234_ This is a desired feature in our study.

## Discussion

The threshold, *t*, in Eq (8) was determined on the basis of the best separation between the good and bad cells, and was dependent upon the power level of X-ray source. In the case of recycling facilities, the power level of X-ray sources can be fixed for scanning all 18650 Li-ion batteries. Therefore, *t* becomes a constant in this case and the proposed sorting would be suited for handling all the 18650 cells without an adjustment of the threshold.

The underlying reasons for a link between the battery degradation and the X-ray imaging of internal structures of batteries may include

1. The material degradation of electrodes and separators leads to less distinctive internal structures of Lithium-ion batteries, and
2. The gradual depletion of electrolytes makes the internal structures of batteries appear blurred in X-ray imaging.

The execution time in Table 3 represents the total execution time of our method. It is in a range of 5 to 6 cells per second, which is the normal processing time of convey belts in a battery recycling facility. Thus, our method has a potential to be applied in real-time battery recycling in a factory setting.

Although the degradation-based sorting accuracy is only 79%, the main purpose of this preliminary study was to prove the feasibility of our method. By using machine learning techniques, the accuracy can be further improved. This is a topic of future work. Previous studies in other fields can be used to support this assertion. In predicting the development of hepatocellular carclinoma [29], the UM regression model and a machine learning algorithm had a c-statistic of 0.6 and 0.64, respectively. In the context of text categorization [30], traditional decision tree generated 67% accuracy, while a machine learning method can achieve 80.5% accuracy.

## Conclusions and future work

The following conclusions can be drawn from this study:

- The degradation-based sorting is feasible via X-ray radiographic scanning and digital image contrast computation with 18650 Lithium-ion batteries at cell level.
- The sorting accuracy of the test cells is about 79%.
- The execution time of our algorithm is about 200 milliseconds, making it well-suited for real-time sorting at battery recycling facilities.
- Our concept of the degradation-based sorting has a potential for a new market of battery reuse via harvesting good used cells.

The future research activities of this study include

- Machine learning [31] will be used to replace the simple linear combination formula in Eq (13). For instance, in a support vector machine non-linear kernel functions are expected to improve the accuracy of the sorting to over 80%.
- Health estimation of multiple cells will be conducted in a pack of 18650 batteries. Each battery pack of a laptop computer usually contains 6 to 12 cells. If the current method could be extended to handle one pack at a time, the recycling efficiency would be significantly improved.
